# FEASIBILITY OF VIRTUAL REALITY TREADMILL TRAINING IN OLDER ADULTS WITH MOBILITY AND FITNESS DEFICITS

**DOI:** 10.1093/geroni/igz038.032

**Published:** 2019-11-08

**Authors:** Steven Castle, Cathy C Lee, Erin H Blanchard, Christian K Roberts, Malcolm J Jones, E T Schroeder

**Affiliations:** 1 University of California, Los Angeles, Los Angeles, California, United States; 2 VA Greater Los Angeles/UCLA, Los Angeles, California, United States; 3 VA Greater Los Angeles Healthcare System, Los Angeles, California, United States; 4 Exercise Physiology, Division of Biokinesiology and Physical Therapy, University of Southern California, Los Angeles, California, United States; 5 Division of Biokinesiology and Physical Therapy, University of Southern California, Los Angeles, California, United States

## Abstract

Only 23.6% of adults and <10% of adults age >75 years meet physical activity (PA) guidelines (aerobic & muscle-strengthening). Health benefit gym memberships has not improved participation. Therefore, Blue Goji developed a Virtual Reality (VR) treadmill to improve PA through fitness gamification with cognitive involvement in postural control to improve balance. Feasibility testing was done with seven older Veterans and one spouse, mean age of 81.3 years who participate in the VA Gerofit program & were near the 50th percentile (by age & gender) in strength, balance and endurance. Even those with lower levels of fitness, balance or chronic conditions (i.e., kyphosis or vision impairment) strongly supported this for enjoyment, benefit, comfort, safety and strongly recommended this (mean of 4.65 out of 5). The Gamification approach supports anti-ageism and intergenerational fitness activities. Further study on the additive effect on exercise intensity and improvement in fitness, balance and cognition is needed.

